# Celiac Disease on the Bed-Side: Embedding Case Finding and Screening in Hospitalized Children

**DOI:** 10.3390/nu15234899

**Published:** 2023-11-23

**Authors:** Angela Pepe, Claudia Mandato, Tiziana Di Leo, Giovanni Boccia, Giulia Lucaroni, Gianluigi Franci, Carolina Mauro, Giuseppe Di Cara, Francesco Valitutti

**Affiliations:** 1 Pediatrics Section, Department of Medicine, Surgery and Dentistry “Scuola Medica Salernitana”, University of Salerno, 84081 Baronissi, Italy; anpepe@unisa.it (A.P.); cmandato@unisa.it (C.M.); glucaroni@unisa.it (G.L.); 2 Clinical Pathology and Biochemistry Unit, University Hospital “San Giovanni di Dio e Ruggi d’Aragona”, 84131 Salerno, Italy; tiziana.dileo@sangiovannieruggi.it; 3 Department of Medicine, Surgery and Dentistry “Scuola Medica Salernitana”, University of Salerno, 84081 Baronissi, Italy; gboccia@unisa.it (G.B.); gfranci@unisa.it (G.F.); 4 Pediatric Unit, Department of Maternal and Child Health, University Hospital “San Giovanni di Dio e Ruggi d’Aragona”, 84131 Salerno, Italy; carolina.mauro@sangiovannieruggi.it; 5 Pediatric Clinic, Department of Surgical and Biomedical Sciences, University of Perugia, 06123 Perugia, Italy; giuseppe.dicara@unipg.it; 6 European Biomedical Research Institute of Salerno (EBRIS), Via De Renzi 50, 84125 Salerno, Italy

**Keywords:** celiac disease, prevalence, case-finding, screening, hospitalized children, malabsorption

## Abstract

Background: Strategies for diagnosing celiac disease (CD) include case-finding and population-screening programs. Case finding consists of testing individuals at increased risk for the disease due to symptoms or associated conditions. Screening programs are widespread campaigns, which definitely perform better in terms of unveiling CD diagnoses but nowadays are still debatable. The global prevalence of CD is around 1% but it almost doubles when considering screening programs among school children. Within this framework, we aimed to estimate the prevalence of CD among hospitalized children in the Pediatric Department of a Southern Italy University Hospital in the period from January 2018 through December 2021. In addition, we attempted to explore, at the time of diagnosis, the prevalence of leading clinical alerts due to malabsorption/malnutrition such as anemia or failure to thrive or due to systemic inflammation/immune dysfunction as hypertransaminasemia and thyroid dysfunction. Methods: Data records of pediatric patients admitted as inpatients and tested by anti-transglutaminase IgA antibodies (TGA-IgA) were retrospectively analyzed. CD was diagnosed according to either 2012 or 2020 ESPGHAN guidelines, depending on the year of diagnosis. CD autoimmunity (CDA) was a wider group defined within our protocol if patients had elevated TGA-IgA on at least one occasion, regardless of anti-endomysial antibodies (EMA-IgA) and without biopsy confirmation. Results: During the observation period, 3608 pediatric patients were admitted and 1320 were screened for CD (median age 5 years, IQR 2–9 years; CD test rate: 36.6% out of all admissions). The available prevalence of newly diagnosed CD was 1.59% (21 patients diagnosed) and the available prevalence of CDA was 3.86% (51 subjects). Among CD patients, underweight/malnourished children accounted for 28.6% (6 out of 21). Conclusions: The estimated prevalence of CD diagnoses within our setting was comparable to the most recent population-screening programs. The estimated prevalence of CDA was even higher. A hospital-admission CD testing during routine blood draws might be a non-invasive, cost-effective and valuable approach to reduce discrepancy of prevalence between case-finding and population-screening programs.

## 1. Introduction

According to the 2020 European Society of Pediatric Gastroenterology, Hepatology and Nutrition (ESPGHAN) diagnostic guidelines, the most accurate and cost-effective method for initial testing for celiac disease (CD) is the combination of total Immunoglobulin A (IgA) and IgA class antibodies against transglutaminase (TGA-IgA). A no-biopsy approach for CD diagnosis is allowed in children with high TGA-IgA values ≥ 10 times the upper limit of normal (ULN) with positive endomysial antibodies (EMA-IgA) in a second serum sample [1].

Based on clinical characteristics, CD can be classified as classical and non-classical [2]. Patients with the classical presentation of CD have the typical symptoms of malabsorption and diarrhea with subsequent malnutrition, failure to thrive and weight loss. The non-classical form mainly presents as extraintestinal manifestation due to systemic inflammation and micronutrient malabsorption such as abnormal liver function, osteoporosis, vitamin deficiencies, anemia, neuropathy, infertility, but also with gastrointestinal symptoms like constipation [3].

Global prevalence of CD is estimated around 1.4% (range 1.1–1.7%) [4]. However, this is not a realistic picture because the vast majority of cases are asymptomatic and undiagnosed.

The epidemiological scenario of CD has radically changed since the introduction of highly sensitive and specific serological tests such as TGA-IgA and EMA-IgA because these allowed population screening and identification of more subtle forms of disease presentation. An important epidemiological finding came from a large multicenter Italian study that identified seven new cases of CD for each established diagnosis [5]. This led to the definition of CD as an underdiagnosed disease and to its representation as an “iceberg” whose tip is represented by the diagnosed subjects and the submerged part by unrecognized or late recognized patients [6,7,8].

An important feature of CD is that clinical manifestations and damage of the intestinal mucosa resolve with a strict and lifelong gluten-free diet which is nowadays the only available treatment for this condition. Gluten-free diet requires strong commitment by patients and their families as well as adequate instructions/follow-up by trained staff (physicians, dieticians, and psychologists) to ensure avoidance of hidden gluten sources, to suggest healthy nutritional choices and to support possible psychological burden of the dietary restriction. This explains why early diagnosis and subsequent follow-up are of utmost importance [9].

Within this background framework, we performed a retrospective study to estimate prevalence of CD among hospitalized children in the Pediatric Department of the “San Giovanni di Dio and Ruggi d’Aragona” University Hospital, Salerno, Italy, between January 2018 and December 2021 and to define the clinical characteristics of patients diagnosed during hospitalization.

## 2. Materials and Methods

### 2.1. Objectives

This study primarily aims to estimate the prevalence of CD in a pediatric inpatient unit. It also examines pediatric patients diagnosed with CD during hospitalization, looking at reasons for admission; moreover, it explores at the time of diagnosis the prevalence of leading clinical alerts due to malabsorption/malnutrition such as anemia or failure to thrive and those due to systemic inflammation/immune dysfunction such as hypertransaminasemia and thyroid dysfunction. Finally, it attempts to compare CD subgroups such as those with symptoms suggestive of CD disease (labelled as Case-Finding group) and those asymptomatic who were diagnosed simply by random testing within laboratory panels as it would happen in screening programs (Screening-Program group).

### 2.2. Study Design

This is a retrospective, single-center study, conducted analyzing the records of patients admitted to the Pediatric Department of the “San Giovanni di Dio and Ruggi d’Aragona” University Hospital in Salerno (Southern Italy) between January 2018 and December 2021.

This study did not imply any change in the diagnostic–therapeutic process to which every patient has been usually subjected.

### 2.3. Subjects and Data

Research through the hospital’s electronic database system was conducted by retrieving all TGA-IgA queries for patients admitted to the Pediatric wards. The medical records of each patient screened by anti-transglutaminase IgA antibodies were examined and a comprehensive database was created ad hoc.

Retrospective data included:—patient demographics (age, gender);—symptoms suggestive of CD on admission such as diarrhea, constipation, headache abdominal pain, poor growth, pallor;—results of routine laboratory tests such as aspartate aminotransferase (AST), alanine aminotransferase (ALT), hemoglobin level (Hb), TSH (thyroid stimulating hormone), and free thyroxine (FT4); where available, results of specific tests such as thyroid autoantibodies, Hepatitis B surface antibody (HbSAb), vitamin D and albumin levels (as proxy for macronutrient and micronutrient malabsorption/malnutrition), total protein levels, Immunoglobulin G (IgG) transglutaminase test, EMA-IgA, and total serum IgA.

### 2.4. Classification Process

CD was diagnosed according to either 2012 or 2020 ESPGHAN guidelines, depending on the year of diagnosis. According to 2012 ESPGHAN guidelines, CD was diagnosed in symptomatic children with TGA-IgA values ≥10 ULN and positive EMA-IgA/HLA (human leukocyte antigens) in a second serum sample or following an intestinal biopsy for those who did not fit these criteria [10]. According to 2020 ESPGHAN guidelines, CD was diagnosed in children with TGA-IgA values ≥10 ULN and positive EMA-IgA in a second serum sample or following an intestinal biopsy for those who did not fit these criteria [2]. CD autoimmunity (CDA) was a wider group defined within our protocol if patients had elevated TGA-IgA on at least one occasion, regardless of anti-endomysial antibodies (EMA-IgA) and without biopsy confirmation.

Patients with CDA, excluding those with an already known CD diagnosis prior to the admission were thus categorized into three groups:

- Admitted patients who showed symptoms suggestive of CD and were subsequently diagnosed with CD or CDA during/following hospital stay (Case-Finding group);
- Admitted patients who were asymptomatic for suggestive symptoms of CD and were diagnosed as CD or CDA by random testing within laboratory panels as it would happen in screening programs (Screening-Program group). The random requests for anti-TGA-IgA and total IgA in this group were part of a calibration protocol for a new antibody kit that was compared with the reference standard antibody kit used for clinical purposes. No extra serum was needed other than the discarded aliquot from the biochemistry lab and that already collected for routine exams.

### 2.5. Statistical Analysis

Statistical analysis was performed using SPSS. Qualitative variables were expressed as percentage, continuous ones as median (interquartile range, IQR). The Shapiro–Wilk test was used to assess normality of data. X^2^ and Mann–Whitney test were performed as appropriate and a *p* < 0.05 was considered significant.

### 2.6. Ethics

This study was approved by the local ethics committee (“Comitato Etico Campania Sud”, regional IRB protocol N. 73 approved on 20 May 2020); informed consent was not obtained due to the retrospective and anonymized approach which did not change the clinical practice nor provoked any privacy leak for confidential data. The research was conducted according to the Declaration of Helsinki regarding the Ethical Principles for Medical Research involving Human Subjects.

## 3. Results

**Figure 1:**
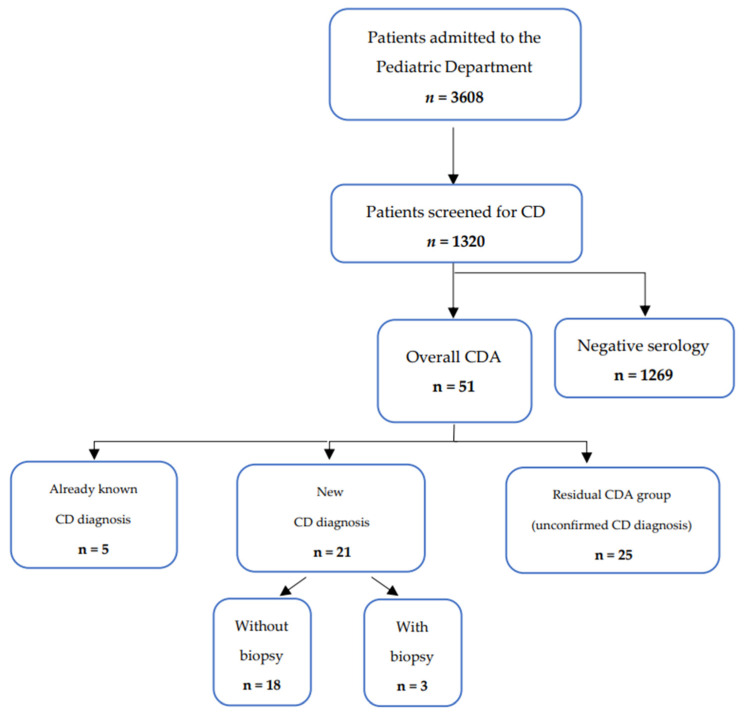
Study population. Legend: CD = celiac disease; CDA = celiac disease autoimmunity.

The study population is described in Figure 1.

During the observation period, 3608 patients were admitted to the Pediatric Department of the “San Giovanni di Dio e Ruggi d’Aragona” University Hospital in Salerno; among them, 1320 patients (M = 691, median age 5 years, IQR 2–9 years) were screened for CD (test rate: 36.6%) and included in the study. No patient had a deficiency in serum levels of IgA.

Overall, there were 51 (3.86%) CDA patients. Among these, 21 (1.59%) patients were confirmed as CD patients. Twenty-five patients (1.89%) had TGA-IgA values < 10 ULN on at least one occasion and negative/transient low-titer EMA-IgA without further biopsy confirmation of CD (residual CDA). Only five children (0.38%) already had a diagnosis of CD when admitted as inpatients.

### 3.1. Celiac Disease Group

Among the twenty-one patients with newly confirmed CD during/following the hospitalization, eighteen patients with TGA-IgA values ≥10 ULN and positive EMA-IgA in a second serum sample were diagnosed with CD without biopsy, whereas three patients not fitting the antibody criteria were biopsy-proven. The median age for CD patients was 4 years (IQR: 2–8).

Demographics of CD patients are resumed in Table 1. Seven patients (33.3%) were asymptomatic for suggestive symptoms of CD and were diagnosed since TGA-IgA were randomly included into laboratory panel. This subset of patients might resemble those identified by means of wide screening programs and thus have been labelled “screening-program” group. Fourteen patients (66.7%) were tested because they showed suggestive symptoms as it happens during active case finding; these latter have been labelled “case-finding” group. Results of laboratory investigations at the initial presentation are shown in Table 2. Six patients (28.6%) had a weight percentage < 5° for age and gender. No patient had a family history of CD. The most common clinical presentations were abdominal pain (23.8%), pallor (23.8%), failure to thrive (19%) and diarrhea (14.3%). Iron deficiency anemia was found in 8 of 21 patients (38%). Moreover, lower ferritin as proxy of decreased micronutrient absorption or increased micronutrient requirement was found in CD compared to controls (*p* < 0.05). No patients had increased ALT levels or abnormal thyroid function. Eight patients (44.4%) had mild elevation in AST levels (1.5–2× ULN). Only two patients were tested for HBsAb, of whom one had a low titer. Similarly, vitamin D levels, tested in only one patient, were found to be deficient. The median value of TGA-IgA was 2116.3 UC (IQR 557.3–4362.2). EMA- IgA positivity at low dilution (1:40) were detected in all patients (100%). The median value of albumin was 4.1 mg/dL (IQR 4.7–4.4).

### 3.2. Celiac Disease Autoimmunity Group

Demographics of residual CDA (unconfirmed CD diagnosis) patients are also shown in Table 1. These twenty-five patients (12 males) had a serum level of TGA-IgA < 10 ULN and no confirmatory diagnosis of CD so far; therefore, this group was still labelled CDA. The prevalence of residual CDA was 1.89%. The median age of this latter group was 5 years (IQR: 3–8).

One patient (4%) had a weight percentile < 5° for age and gender and 4 patients (16%) had a weight percentile > 95°. No patient of CDA group had a family history of CD. The most common clinical presentation at the time of hospitalization was abdominal pain (16%). No patients had abnormal thyroid function. One patient (4%) had hypertransaminasemia (transaminase levels > 10 ULN); in this case, autoimmune hepatitis was diagnosed.

The median value of TGA-IgA in CDA was 55.1 UC (IQR 31.2–68.7). Four patients (16.7%) had a positivity in EMA-IgA values (also in Table 2), but the dilution titer was very low and not confirmed on follow-up blood tests despite normal gluten intake. Two CDA subjects (8%) were tested for HbSAb and a normal titer was found.

A lower ferritin was found in CDA compared to controls (*p* < 0.05).

CDA patients were included in a follow-up program which is still ongoing.

### 3.3. Patients Already Diagnosed with CD

Among the 1320 patients tested for CD during the admission to the Pediatric Department, only 5 patients (0.38%) already had a diagnosis of CD; two of them had still elevated levels of TGA-IgA (>10 ULN) with positive EMA-IgA and iron deficiency anemia. Clinical data, available for 4 out of 5 patients, showed that two patients had been diagnosed with CD less than a year earlier and two others had been on a gluten-free diet for more than a year.

### 3.4. Comparison between Clinical Characteristics of CD and Residual CDA Group

Regarding the clinical characteristics of the main study groups, the most common symptom of both CD and residual CDA was abdominal pain (23.8% and 16% in CD and CDA group, respectively). In addition, 6 patients (28.6%) from the CD group and 1 patient (4%) from the residual CDA group had a weight percentage < 5° for age (*p* = 0.0576); 4 patients with residual CDA (16%) were obese, compared to 2 (9.5%) obese patients of the CD group (*p* = 0.8335) (Table 3). No patients between the two groups had a family history of CD.

**Figure 2:**
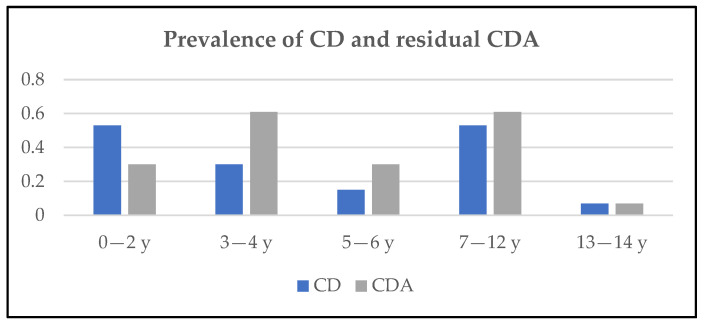
Age distribution of CD and residual CDA cases.

The analysis of CD and residual CDA distribution in the different age groups showed that CD had an overlapping prevalence (0.53%) between the 0–2 and 7–12 age groups while CDA had a prevalence of 0.61% in both the 3–4 and 7–12 age groups (Figure 2). However, in screening-detected patients, none of the considered clinical parameters were predictive of CD.

## 4. Discussion

To the best of our knowledge, this is the first study attempting to describe the prevalence of CD among hospitalized children. Our study aims to retrospectively assess the prevalence of CD in a cohort of hospitalized children during the years 2018–2021 at the Department of Pediatrics of the University Hospital of Salerno, where there is no default mass-screening protocol in place for all children admitted. Notwithstanding this, we detected a CD prevalence of 1.59% which overlaps the most recent population-screening programs (prevalence: 1.65%) among school children [11]. When also considering those children with a CD diagnosis before the admission, this prevalence rises to 1.96%.

Data analysis showed that CD was diagnosed without biopsy in 18 and was instead biopsy-proven in 3 children. Among the 21 diagnosed CD children, 14 were diagnosed by a so-called case-finding approach and seven by random serology request to the laboratory which could somewhat resemble a screening program.

Considering all tested patients with symptoms suggestive of CD, the estimated prevalence of diagnosis by case finding is 4.05%. On the other hand, the prevalence of CD by screening program among all tested asymptomatic patients is 0.72%. No statistically significant difference as regards the mean values of TGA-IgA between these latter subgroups were found.

A remarkable increase in the diagnosed cases of CD has been registered in recent decades, partly due to the development of serological diagnostic tests, partly to possible environmental changes such as variations in the quantity and quality of ingested gluten and infant nutrition influencing disease onset [8,12,13].

The prevalence of CD varies with gender, age and geographical location: the estimated prevalence for CD was 0.4% in South America, 0.5% in Africa and North America, 0.6% in Asia, and 0.8% in Europe and Oceania, with a higher prevalence in females and children [4].

The prevalence of CD in Italy has increased over the years. A 2020 National Health Service [14] report reveals that on 31 December 2019, there were 233,147 celiac patients in Italy, 66% of whom were female. New diagnoses in 2020 were lower (7729) than in 2019 (11,179), probably due to the diagnostic delay occurring during Severe Acute Respiratory Syndrome Coronavirus 2 pandemic [15,16].

Considering the Italian population (58,851,000 people), and the estimated CD prevalence in Italy (1%), there could be more than 350,000 people affected by CD but not diagnosed, with huge costs for the national health system [17]. This corresponds to the submerged part of the so-called celiac “iceberg” [5,18]. This is the most insidious and dangerous aspect of the disease because most cases are not promptly diagnosed.

Although CD meets the criteria for a population screening according to the World Health Organization [19], many questions remain about its applicability. For example, the age of onset is variable; therefore, it is difficult to establish what the ideal age for screening is and how positive/negative predictive values perform in this setting; in addition, screening could also identify doubtful cases that would raise the question about the type of follow-up that should be implemented. The diagnostic strategies for CD are substantially of two distinct classes: population/mass screening or case finding, i.e., testing individuals who are at increased risk for CD primarily due to the presence of symptoms or conditions associated such as autoimmune disorders [14,20]. The jury is still out as regards the best diagnostic strategy (mass screening vs. case finding) for CD [21].

Case finding is certainly cheaper than a screening program, but it is not as effective in identifying the high number of asymptomatic patients. However, the implementation of population screening is currently under discussion for several reasons: costs for health systems, doubts about the best age for screening, uncertainty about the evolution of mild symptomatic cases and about follow-ups for residual CDA, difficulty for asymptomatic patients to comply with a strict gluten-free diet.

Despite these issues, a screening approach would have some impact on mortality and morbidity, as the lack or delay of diagnosis has several effects on the increasing risk of developing cancer or comorbidities such as osteoporosis [22].

Our data show a higher prevalence of CD among hospitalized children than the acknowledged national data of 1% and an overlapping prevalence with the recent screening study in Italian school children. Nevertheless, when also including patients diagnosed with CD prior to admission, our prevalence is even higher (1.96%). This might be partly attributable to some selection bias, since patients with undiagnosed CD may be more susceptible to severe forms of infections which require hospital admission [23,24]. In our setting, most diagnosed cases still resemble case-finding cases since 62% showed, at the time of the admission, signs and symptoms attributable to CD despite having been hospitalized for acute diseases. Six CD patients out of twenty-one (28.6%) showed a weight percentile lower than 5°, addressing once more the importance of inpatient growth/nutritional assessment as a rescue opportunity for primary care well-being checks [25]. Moreover, we found lower ferritin levels in both CD and residual CDA, perhaps suggesting a decreased micronutrient absorption or increased micronutrient requirement in these groups.

Consequently, empowering a hospital program to test all admitted children at a very low-threshold for CD leads to an increase in the number of diagnoses. The same applies to possible hospital-based wider screening programs for inpatients, albeit a cost-effective projection is not possible given our data.

A previous study conducted in Italy analyzed data about the clinical presentation of CD in children diagnosed in an outpatient setting between 1990 and 2020 [26]: in the period of interest, recurrent abdominal pain was described as prevailing (51%), followed by diarrhea (30%); failure to thrive had a prevalence of 21% in CD patients. The analysis of the medical records from our database showed that abdominal pain was the most common symptom in both CD and residual CDA subjects. In addition, more than 40% of CD patients had iron deficiency anemia. This finding is compatible with a recent meta-analysis [15], which highlights that the prevalence of anemia among CD patients varies widely between studies, ranging from 12% to up to 85%.

As regards the case distribution, the highest prevalence was registered in the 0–2 and 7–12 age groups, and the median age at diagnosis was 4 years, slightly lower than in a previous study conducted in the Mediterranean area showing a median age of 5 years old at diagnosis [27].

Another interesting finding from our cohort is that a disease with such a high prevalence led to hospitalization of only five already diagnosed patients over 4 years (0.38%). Contrarily, a study by Canova et al. disclosed that CD subjects had a higher risk of hospitalization and medication use compared to the general population, even five or more years after diagnosis [24]. Among our already diagnosed CD patients, two out of five patients still had elevated TGA-IgA levels more than a year after diagnosis, which may indicate poor compliance with the gluten-free diet. Nevertheless, given our data it is not possible to speculate whether a previous diagnosis of CD and perhaps a good dietary compliance coupled to a well-balanced macronutrient/micronutrient intake is of any relevance in preventing hospital admission. In addition, due to the retrospective design of the study, we are unaware whether some of the negative children, of the CD patients or of CDA children, have received supplementation with probiotics which might hypothetically play a role in the progression and clinical presentation of the disease CD [28,29,30,31,32].

Some limitations of our work must be acknowledged: this is a retrospective study where CD prevalence might be either slightly underestimated or, more possibly, overestimated due to selection bias. In fact, only 36.6% of patients admitted to the Pediatric Department for acute illnesses were tested for CD and a proper screening program for all inpatients has not been carried out.

Although interpreting the results of our study should take into account these limitations, these results regarding 1320 hospitalized children underscore the feasibility of a hospital-based CD screening for admitted patients with less organizational burden than proper population-screening programs. Moreover, this work widens the perspective on current CD clinical presentation and epidemiology.

## 5. Conclusions

In conclusion, albeit limited to those who had been tested, the prevalence of overall CDA in our hospitalized cohort was high (3.86%), with a prevalence of confirmed diagnoses (1.59%) comparable to the most recent population-screening programs [11]. An empowered case finding—especially looking at subtle signs of malabsorption/malnutrition—is always advisable in all pediatric hospital admissions. Nevertheless, a default screening program during routine blood draws for hospitalized children might be a valuable approach to reduce discrepancy of prevalence between outpatient case-finding and population-screening programs.

## Figures and Tables

**Table 1:** Demographics of the CD group and of the residual CDA group. ns—non-significant.

Gender (%)	CD	Residual CDA	*p* Value
Male	8 (38.1%)	12 (48%)	*p*: ns
Female	13 (61.9%)	13 (52%)	*p*: ns
**Median age, years (IQR)**	4 (2–8)	5 (3–8)	*p*: ns
**Gestational age**			
Term	20 (95%)	23 (92%)	*p*: ns
Preterm	1 (5%)	2 (8%)	*p*: ns
**History of breastfeeding**	12 (57.1%)	17 (68%)	*p*: ns
**Weight percentile at admission *n* (%)**			
<5°	6 (28.6%)	1 (4%)	*p*: ns
5–10°	1 (4.8%)	1 (4%)	*p*: ns
10–25°	2 (9.5%)	0	*p*: ns
25–50°	4 (19.0%)	7 (28%)	*p*: ns
50–75°	5 (23.8%)	2 (8%)	*p*: ns
75–90°	1 (4.8%)	5 (20%)	*p*: ns
90–95°	0	5 (20%)	*p*: ns
>95°	2 (9.5%)	4 (16%)	*p*: ns
**Age at presentation**			*p*: ns
0–2	7 (33.3%)	4 (16%)	*p*: ns
3–4	4 (19.0%)	8 (32%)	*p*: ns
5–6	2 (9.5%)	4 (16%)	*p*: ns
7–12	7 (33.3%)	8 (32%)	*p*: ns
13–14	1 (4.8%)	1 (4%)	*p*: ns

**Table 2:** Laboratory findings in the three groups. Values are presented as median (interquartile range, IQR) except for EMA IgA where positive cases are shown as numbers. * refers to comparison between CD and CDA. # refers to comparison between CD and negative TGA IgA. § refers to comparison between CDA and negative TGA IgA. ns—non-significant.

Laboratory Investigation	CD Group (*n* = 21)	CDA Group (*n* = 25)	Negative TGA IgA (*n* = 1269)	*p* Value
Hb (g/dL)	12.2 (10.9–13.5)	12.9 (11.7–13.4)	12.6 (11.7–13.4)	*p*: ns
ALT (U/L)	19 (16–22)	17 (15–25)	17 (14–23)	*p*: ns
AST (U/L)	35 (26–44)	30 (27–36)	31 (25–42)	*p*: ns
Ferritin (ng/mL)	18.6 (12.4–25.2)	16.8 (10.1–45.2)	34.6 (20.9–63.3)	*p* < 0.05 (#,§); *p*: ns *
TSH µIU/mL	2.08 (1.15–2.95)	1.84 (1.02–2.73)	1.98 (1.35–2.86)	*p*: ns
FT4 ng/dL	1.06 (0.93–1.34)	1.08 (1–07–1.25)	1.16 (1–1.32)	*p*: ns
TGA-IgA	2116.3 (557.3–4362.2)	55.1 (31.2–68.7)	1.9 (1.9–1.9)	*p* < 0.05 (#,§,*)
Total protein (mg/dL)	6.6 (5.9–7.3)	6.8 (6.2–7.1)	6.7 (5.8–7.0)	*p*: ns
Albumin (mg/dL)	4.1 (3.7–4.4)	3.8 (3.5–4.3)	4.0 (3.5–4.4)	*p*: ns
Positive EMA-IgA	21	4	0	*p* < 0.05 (#,§,*)

**Table 3:** Comparison of clinical features between CD and CDA group. ns—non-significant.

Clinical Features	CD Patients *n* = 21 (%)	Residual CDA Patients *n* = 25 (%)	*p* Value
Diarrhea	3 (14.3%)	2 (8%)	*p*: ns
Constipation	1 (4.8%)	3 (12%)	*p*: ns
Pallor	5 (23.8%)	4 (16%)	*p*: ns
Failure to thrive	4 (19%)	0	*p*: ns
Abdominal pain	5 (23.8%)	4 (16%)	*p*: ns
Headache	3 (14.3%)	0	*p*: ns
Arthralgias	3 (14.3%)	2 (8%)	*p*: ns
Oral aphthosis	0	1 (4%)	*p*: ns
Family history of CD	0	0	*p*: ns
Weight percentile < 5°	6 (28.6%)	1 (4%)	*p*: ns
Weight percentile 5–95°	13 (61.9%)	20 (80%)	*p*: ns
Weight percentile > 95%	2 (9.5%)	4 (16%)	*p*: ns

## Data Availability

Data can be obtained from the authors upon request.

## Abbreviations

ALT: alanine aminotransferase; AST: aspartate aminotransferase; CD: Celiac Disease; CDA: Celiac Disease autoimmunity; EMA-IgA: endomysial antibodies; ESPGHAN: European Society of Pediatric Gastroenterology, Hepatology and Nutrition; FT4: free thyroxine; Hb: hemoglobin; HbSAb: Hepatitis B surface antibody; HLA: Human leukocyte antigens; Ig: Immunoglobulin; IQR: Interquartile Range; TGA-IgA: Immunoglobulin A class antibodies against transglutaminase; TSH: thyroid stimulating hormone; ULN: upper limit of normal.
